# Risk factors associated with limited functional status among out-of-hospital patients 30 days and one year after a diagnosis of COVID-19: a cohort study

**DOI:** 10.1038/s41598-023-30674-0

**Published:** 2023-03-03

**Authors:** Larissa Laskovski, Josiane Marques Felcar, Michelle Moreira Abujamra Fillis, Celita Salmaso Trelha

**Affiliations:** 1 Londrina State University, Londrina, Paraná, Brazil; 2 North Paraná State University, Jacarezinho, Paraná, Brazil

**Keywords:** Diseases, Health care, Risk factors

## Abstract

Some people experience indefinitely persistent and disabling symptoms after acute COVID-19, even those who have not been hospitalized. The purpose of this study was to analyze the long-term health consequences at 30 days and one year among people who were not hospitalized after a diagnosis of COVID-19 and to analyze which variables predict limitations in functional status. This is a prospective cohort study with non-hospitalized adults infected with SARS-CoV-2 in the city of Londrina. After 30 days and one year of the acute symptoms of COVID-19, participants received the questionnaire through a social media that consisted of sociodemographic data and data on functionality through the Post-COVID Functional State Scale (PCFS)—the primary outcome of the study "presence of functional status limitation" was grouped into without functional status limitation (value: zero) and with functional limitation (value 1 to 4), fatigue through of the Fatigue Severity Scale (FSS) and dyspnea using the modified Borg scale. In the statistical analysis, multivariable analysis was performed. Statistical significance was set to 5%. Of 140 individuals analyzed, 103 (73.6%) were female with a median age of 35.5 (27–46) years. One year after the diagnosis of COVID-19, 44.3% had at least one self-reported symptom: memory loss (13.6%), gloominess (8.6%), anosmia (7.9%), body pain (7.1%), ageusia (7%), headache (6.4%), and cough (3.6%). According to the FSS and modified Borg scale 42.9% reported fatigue and 18.6% reported dyspnea, respectively. As for functionality, 40.7% reported some limitation, being 24.3% negligible functional limitation, 14.3% slight and 2.1% moderate according to the PCFS. There was a univariate association between the presence of limitation in the functional status with the female sex, diagnosis of anxiety and depression, presence of persistent symptoms after one year, fatigue and dyspnea. In the multivariable analysis, the predictor variables for functional status limitation were female sex, diagnosis of anxiety/depression, presence of at least one persistent symptom and fatigue one year after the diagnosis of COVID-19. One year after the disease, the patients presented functional limitation according to the PCFS, even without hospitalization. Risk factors associated with functional limitation include female sex, presence of fatigue, anxiety and depression, and at least one persistent symptom after one year of COVID-19 diagnosis.

## Introduction

The clinical presentation and consequences of coronavirus infection range from asymptomatic cases to death. Likewise, the duration of COVID-19 varies among afflicted individuals; while some recover quickly, others experience persistent and often disabling symptoms^1^ that impact everyday functioning^2^. Post-COVID-19 condition is defined as the illness that occurs in people who have a history of probable or confirmed SARS-CoV-2 virus infection; usually within three months from the onset of COVID-19, with symptoms and effects that last for at least two months^3^. Prolonged symptoms following infection with SARS-CoV-2 that are not explained by an alternative diagnosis are called by the term “long COVID”^3,4^.

A retrospective cohort study to determine symptoms that are associated with confirmed SARS-CoV-2 infection beyond 12 weeks in non-hospitalized adults and the risk factors associated with developing persistent symptoms. A total of 62 symptoms were significantly associated with SARS-CoV-2 infection after 12 weeks. The largest were for anosmia, hair loss, sneezing, ejaculation difficulty and reduced libido. Among the cohort of patients infected with SARS-CoV-2, risk factors for long COVID included female sex, belonging to an ethnic minority, socioeconomic deprivation, smoking, obesity and a wide range of comorbidities. The risk of developing long COVID was also found to be increased along a gradient of decreasing age. The study concludes that SARS-CoV-2 infection is associated with a plethora of symptoms that are associated with a range of sociodemographic and clinical risk factors^2^.

The post-COVID-19 condition is complex and multidimensional and involves many organ systems. Different post-COVID-19 condition phenotypes might exist, although exact causes, management, and outcomes are not known^5^. The most prevalent symptoms, such as fatigue, shortness of breath, and cognitive dysfunction, which generally have an effect on everyday functioning such as work or household chores^3,5^. Another study says that women, malignant disease and hypertension presented a risk factor for greater functional impairment in long COVID^6^. A systematic review including non-hospitalized patients with mild COVID-19 infection found that symptoms of mild COVID-19 persist after 3 weeks in a third of these patients and can have major consequences for work and daily functioning^7^.

On the other hand, acute phase care takes place in strict isolation, which favors the reduction of individuals' mobility regardless of the severity of the clinical condition^8^. The Ministry of Health, a government agency in Brazil, determined during the pandemic that patients with COVID-19 would be isolated for a period of 14 days and that this period could be extended for up to the same period as prescribed by a doctor^9^. In Brazil, despite differences in restrictive measures between states, in general, this action was adopted throughout the country. In the city of Londrina - Paraná and region, government attitudes were characterized by a period of great control, such as the closing of commercial establishments and suspension of academic activities, maintaining only essential services, from March 22, 2020, which lasted until May 23, 2020^10^. From this date, municipal measures declared the gradual return of commercial activities, maintaining health and distancing measures.

While many researchers have focused on individuals who required hospitalization^8,11,12^, the majority of COVID-19 patients are not hospitalized^13^. Even in non-hospitalized COVID-19 patients, it may take time to return to pre-illness health status^13^. In this context, the purpose of this study was to analyze the long-term health consequences at 30 days and one year among people who were not hospitalized after a diagnosis of COVID-19 and to analyze which variables predict limitations in functional status.

## Methodology

This was a prospective cohort study, part of a larger project entitled "Clinical functional assessment and quality of life of patients one, two, six, and 12 months after a diagnosis of SARS-CoV-2 infection in a city of a municipality in South America with more than half a million inhabitants”. This study was developed and conducted in partnership between a State University and the Municipal Health Department. This research was authorized by the Municipal Health Department and approved by an Institutional Review Board (IRB) - no. 36782620.0.000.5231. Additionally, the research was approved by the Ethics Committee for Research Involving Humans of the State University of Londrina under protocol 4.235.042.

The authors confirm that all research was performed in accordance with relevant regulations and declare that informed consent was obtained from all participants. In the form, the participant was instructed that “By clicking the button below, you agree to participate in the research under the terms of this free and informed consent form. If you do not agree to participate, just close this page in your browser.” All participants received information about the research and answered the questionnaire only after agreeing to participate in accordance with the free and informed consent form. This study followed the STROBE statement^14^.

Participants were recruited through a survey carried out on the official platform of the state notification system of a South American state called Notify COVID-19, a single platform for the compulsory notification of suspected and confirmed cases of COVID-19 in all municipalities. A form was filled out by health professionals for all patients with flu-like syndrome and contained identification data, clinical data (signs and symptoms), presence of comorbidities or risk factors, hospitalization information, medications, imaging tests, laboratory data of the patient, classification of the notification (suspected, discarded or confirmed) and classification criteria (laboratory or clinical/epidemiological criteria).

**Figure 1:**
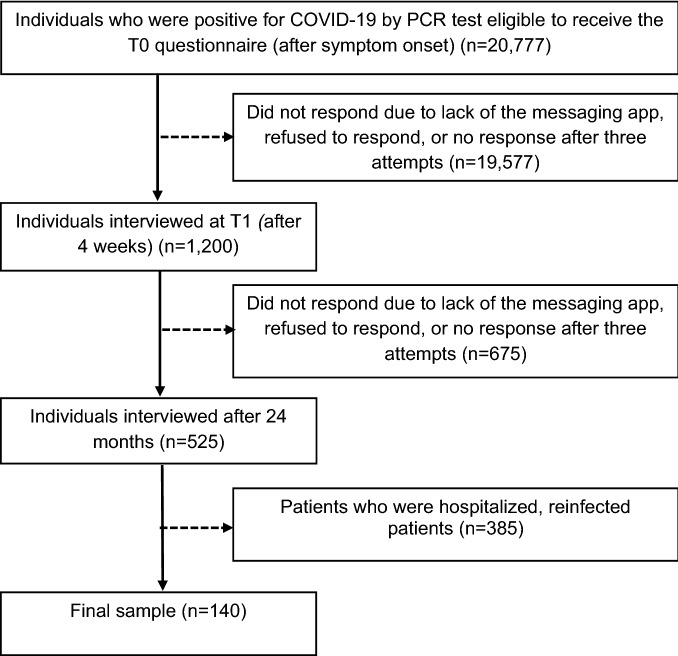
Participant flowchart.

All individuals with a positive diagnosis by an RT–PCR molecular test for SARS-CoV-2 aged 18 years or over who agreed to participate were included in this study. Individuals with an unknown etiological agent, those who were unable to be reached, those in an institution, older people in long-term care institutions, people who had difficulty using digital technology or accessing the internet and patients who were hospitalized were excluded (see Fig. 1).

Data collection was carried out from October 2020 to January 2022. Participants received the questionnaire (Google Forms) thirty days and one year after COVID-19 symptom onset; the questionnaire consisted of sociodemographic data, anthropometric data (sex, age) and symptomatic manifestations caused by COVID-19 in adults^15^ and comorbidities. To estimate the number of participants in this study, the G*Power 3.1.9.7 (University of Dusseldorf, Germany) Z test was used for logistic regression, with binomial distribution and input effect size as two probabilities. Sample size was calculated using confidence intervals (CIs) = 95%, significance = 5% and power = 95%, which indicated 106 patients. Considering a loss of 20%, 127 patients would be needed.

The primary outcome of this study was Post-COVID-19 Functional Status (PCFS) Scale grade^16^, which was used to assess functional status. The PCFS scale covers the full range of functional outcomes, as it focuses on limitations in daily tasks/activities at home or at work/school, as well as changes in lifestyle. This questionnaire can be self-administered. The scale has 6 grading possibilities: 0 (zero: no limitation), 4 (four: severe functional limitation) and 5 (five: death). The scale can be applied at hospital discharge and during outpatient follow-up to assess and monitor functional status.

The Fatigue Severity Scale (FSS)^17^ was also used, which is a questionnaire containing nine statements for which the patient chooses a number from one to seven that best describes their agreement with each statement. Number one indicates that the individual completely disagrees, number seven indicates that the individual completely agrees, and number four indicates that the patient neither agrees nor disagrees with the statement. The total number of points can vary from nine to 63, and values ​​equal to or greater than 28 are indicative of the presence of fatigue^18^. For dyspnea, the modified Borg scale^19^ was used.

During the survey, patients were asked whether any relevant health events occurred during one year of follow-up and whether any new medical diagnoses were made during this period. Patients with COVID-19 reinfection were excluded from this study.

After completing the questionnaires, the participant received a booklet of guidelines and exercises based on material prepared by the World Health Organization (WHO) in 2021^20^, which provided support for self-managed rehabilitation after SARS-CoV-2. The booklet contained information about dyspnea management, physical exercise, guidance on voice problems, difficulties in activities of daily living, cognitive changes, mood and stress changes, and emergency contacts.

### Statistical analysis

The Shapiro–Wilk test was used to analyze the normality in the distribution of numeric variables. As they did not reach their assumptions, they were described as median and their quartiles (1º; 3º). Categorical variables were presented through absolute and relative frequency**.**

To compare numerical variables between groups (gender) or comparisons between groups (dyspnea, fatigue, functional limitations and persistent symptoms 30 days and one year after COVID-19 diagnosis) the Mann–Whitney test was used and to assess the association between categorical variables, chi-square test (with or without Yates correction) was used.

For the analysis of associated factors, the dependent variable "presence of functional status limitation" was grouped into without functional status limitation (value of zero) and with functional limitation (value of 1 to 4)^16^.

A univariate analysis using cross tabulation and chi-square tests (with Yates continuity correction whenever indicated) was employed to establish an association between the with/without functional limitation categories one year after the diagnosis and the predictor variables (sex, obesity, diagnosis of chronic disease, fatigue, persistent symptoms, dyspnea at 30 days and one year).

Statistically significant values ​​in these first analyses were used in the stepwise logistic regression model (forward likelihood ratio) with adjusted odds ratios and 95% CIs set to express their magnitude^21^.

To proceed with the multivariable analysis, the following assumptions were made: a) the omnibus chi-square test of model coefficients was used to verify whether the set of variables improved the prediction of the log odds; b) the Nagelkerke R^2^ investigated whether these independent variables could explain low variance in the sum; and c) the Hosmer–Lemeshow goodness-of-fit test was used to evaluate whether the observed values ​​were close to those expected^22^.

Evaluation of the accuracy of the model prediction was performed to check the model’s ability to accurately classify the presence/absence of functional limitations one year after the diagnosis. For the stepwise analysis, the probabilities for variable entry and removal from the model were set between 0.05 and 0.10^22^. Statistical significance was set at 5%, and all analyses were performed with MedCalc® statistical software version 20.014 (Ostend, Belgium) and IBM SPSS® version 27 (Armonk, NY, USA).

### Ethics approval and consent to participate

The research was authorized by the Municipal Health Department and approved by the IRB #36782620.0.000.5231. All participants received information about the research and answered the questionnaire only after agreeing to the research in accordance with the Informed Consent Form. Ethical approval by CAAE: 36782620.0.0000.5231 and ethics protocol reference number: 4.235.042. Ethics Committee in Research with Human Beings of the Londrina State University. The authors confirm that all research was performed in accordance with relevant regulations, and confirm that informed consent was obtained from all participants.

## Results

Among the 140 individuals analyzed, 103 (73.6%) were female, with a median age of 35.5 (27–46) years. Of these, 55% reported having a diagnosis of at least one health condition, the most frequent being obesity (27.9%), anxiety/depression (25.7%), systemic arterial hypertension (10%) and diabetes mellitus (5.7%). The general characteristics of the sample are shown in greater detail in Table 1.

**Table 1:** Participant characteristics.

Variable	Total N = 140	Female N = 103	Male N = 37	*P*
Age*(years)	35.5 [27–46]	34 [26–48]	34[31–46]	0.208
**Chronic diseases (N; %)**				
No	63 (45%)	44 (42.7%)	19 (51.4%)	
Yes	77 (55%)	59 (57.3%)	18 (48.6%)	0.375
**SAH**				
No	126 (90%)	94 (91.3%)	32 (86.5%)	
Yes	14 (10%)	9 (8.7%)	5 (13.5%)	0.406
**Diabetes mellitus**				
No	132 (94.3%)	98 (95.1%)	34 (91.9%)	
Yes	8 (5.7%)	5 (4.9%)	3 (8.1%)	0.465
**Obesity (BMI ≥ 30)**				
No	101 (72.1%)	73 (70.9%)	28 (75.7%)	
Yes	39 (27.9%)	30 (29.1%)	9 (24.3%)	0.576
**Anxiety/depression**				
No	104 (74.3%)	72 (69.9%)	32 (86.5%)	
Yes	36 (25.7%)	31(26.5%)	5 (13.5%)	0.07

*Source*: Prepared by the authors (2022).

Chart legend: *Md [1st and 3rd q]; SAH: systemic arterial hypertension; BMI: body mass index.

For comparisons between groups, the Mann‒Whitney U test or chi-square were used.

One year after a diagnosis of COVID-19, 44.3% had at least one self-reported symptom: memory loss (13.6%), gloominess (8.6%), anosmia (7.9%), body pain (7.1%), ageusia (7%), headache (6.4%), and cough (3.6%). In addition, 42.9% reported fatigue, and 18.6% reported dyspnea, according to the FSS and Modified Borg scale, respectively (Table 2).

**Table 2:** Dyspnea, fatigue, functional limitations and persistent symptoms 30 days and one year after COVID-19 diagnosis.

Variable	30 days	1 year	*P*
**Dyspnea (N; %)**			≤ 0.001**
No	101(72.1%)	114 (81.4%)	
Yes	39 (27.9%)	26 (18.6%)	
**Fatigue** (N; %)			≤ 0.001**
No	65 (46.4%)	80 (57.1%)	
Yes	75 (53.6%)	60 (42.9%)	
Fatigue severity scale (FSS)*	30 [16.3–44]	22 [12–38]	≤ 0.001**
**Functional limitations** (N;%)			≤ 0.001**
No	65 (46.4%)	83 (59.3%)	
Yes	75 (53.5%)	57 (40.7%)	
**PCFS scale** (N; %)			≤ 0.001**
Grade 0: No limitation	65 (46.4%)	83 (59.3%)	
Grade 1: negligible limitation	32 (22.9%)	34 (24.3%)	
Grade 2: slight limitation	35 (25%)	20 (14.3%)	
Grade 3: moderate limitation	7 (5%)	3 (2.1%)	
Grade 4: severe limitation	1 (0.1%)	0	
**Persistent symptoms**			≤ 0.001**
No	51(36.4%)	78 (55.7%)	
Yes	89 (63.6%)	62 (44.3%)	

*Source*: Prepared by the authors (2022).

Chart legend: *Md [1st and 3rd q]; **P ≤ 0.05. For comparisons between groups, the independent t test or the Mann‒Whitney U test was used.

Regarding functionality, 40.7% reported some limitation, with 24.3% having very negligible functional limitations, 14.3% having slight functional limitations and 2.1% having moderate functional limitations according to the PCFS Scale. There was a univariate association between the presence of limitations in functional status and female sex, diagnosis of anxiety and depression, presence of persistent symptoms after one year, fatigue and dyspnea (Table 3).

**Table 3:** Univariate association between functional status limitations after one year and sex, comorbidities, persistent symptoms, fatigue, and dyspnea.

	Functional limitation (n = 57)	No functional limitation (n = 83)	*P*
**Sex**			≤ 0.001*
Female	52 (91.2%)	51 (61.4%)	
Male	5 (8.8%)	32 (38.6%)	
**Chronic diseases**			0.59
Yes	24 (42.1%)	30 (36.1%)	
No	33 (59.9%)	53 (63.9%)	
**Anxiety/depression**			≤ 0.001*
Yes	26 (45.6%)	5 (6%)	
No	31 (54.4%)	78 (94%)	
**Obesity (BMI)**			0.54
Yes	18 (31.6%)	21 (25.3%)	
No	39 (68.4%)	62 (74.7%)	
**Dyspnea after 30 days**			0.019*
Yes	22 (38.6%)	66 (79.5%)	
No	35 (61.4%)	17 (20.5%)	
**Dyspnea after 1 year**			≤ 0.001*
Yes	19 (33.3%)	7 (8.4%)	
No	38 (66.7%)	76 (91.6%)	
**Fatigue after 30 days**			0.01*
Yes	38 (66.7%)	37 (44.6%)	
No	19 (33.3%)	46 (55.4%)	
**Fatigue after 1 year**			≤ 0.001*
Yes	39 (68.4%)	62 (74.7%)	
No	19 (33.3%)	21 (25.3%)	
**Presence of persistent symptoms after 1 year**			≤ 0.001*
Yes	38 (66.7%)	24 (28.9%)	
No	19 (33.3%)	59 (71.1%)	

*Source*: Prepared by the authors (2022).

Chart legend: BMI: body mass index, *P ≤ 0.05. For comparisons between groups, the independent t test or the Mann‒Whitney U test was used.

For the multivariable analysis, nine independent variables entered the stepwise logistic regression model (Table 3). Only sex, anxiety/depression, dyspnea after 30 days, dyspnea after one year, fatigue after 30 days, fatigue after one year, and persistent symptoms after one year remained associated with the dependent variable functional limitation after one year (omnibus tests of model coefficients *P* < 0.001).

In the multivariable analysis, the predictor variables for functional status limitation were female sex, diagnosis of anxiety/depression, presence of at least one persistent symptom and fatigue one year after the diagnosis of COVID-19 (Table 4).

**Table 4:** Multivariable analysis of functional limitations after one year and predictor variables.

	OR (95% CI)	*P*
Constant		< 0.001*
Sex	8.31(1.9;36.01)	0.005*
Anxiety/depression	23.94(6.0;95.44)	< 0.001*
Dyspnea after 30 days	0.4(0.08–1.81)	0.13
Dyspnea after 1 year	4.07(0.79;20.91)	0.09
Fatigue after 30 days	0.43 (0.12;1.56)	0.201
Fatigue after 1 year	6.01(1.82;19.81)	0.003*
Persistent symptoms after 1 year	4.42 (1.45;13.41)	0.009*

*Source*: Prepared by the authors (2022).

Chart legend: OR: odds ratio; CI: confidence interval; *P ≤ 0.05.

## Discussion

The aim of this study was to analyze the long-term health consequences at 30 days and one year among people who were not hospitalized after a diagnosis of COVID-19 and to analyze which variables predict limitations in functional status. A total of 53.5% of patients had functional limitations according to the PCFS Scale 30 days after a diagnosis of COVID-19 and 40.7% after one year. The predictor variables were anxiety/depression, sex, presence of fatigue and persistent symptoms after one year.

Pant et al*.*^23^ reported that 83% of 106 patients who recovered from COVID-19 did not require hospitalization. In this study, the prevalence of some degree of functional limitation was observed among 46 (43.4%) patients, with 27.3% of patients presenting negligible functional limitations (PCFS grade 1) and 13 presenting (12.3%) slight (PCFS grade 2) and moderate (PCFS grade 3) functional limitations. Only 2 (1.9%) had severe functional limitations (PCFS grade 4).

The PCFS Scale is a tool developed by Klok et al. to follow the course of symptoms and their impact on functional status among COVID-19^24^ survivors. The PCFS Scale is easy to apply and can be self-administered; for this reason, the researchers chose to use it with the interest of determining the functional status of individuals who were not hospitalized due to COVID-19. The PCFS Scale has proven to be a simple, reproducible and low-cost method for monitoring patient care measures after the acute phase of COVID-19^23^.

Most long COVID studies focus on patients with severe symptoms after hospitalization or those seen in outpatient clinics or specialized units, which can overestimate the frequency and severity of symptoms. Although severe COVID-19 is associated with a high risk of long COVID, patients with asymptomatic or mild illness may also have it. Long COVID occurs in patients with severe symptoms, but it has also been reported regardless of the severity of the acute phase and hospitalization^2,23–29^.

A substantial number of COVID-19 survivors have persistent symptoms with subsequent progression to decline in functional status^13,16,23,24,30^. As in this study, fatigue and dyspnea were reported even among those post-COVID-19 patients who did not require hospital admission^13,16^. These symptoms have a long-term impact on the physical, mental, social and cognitive function of those infected with COVID-19, causing a decline in functional status^24^.

Many published papers have discussed persistent symptoms and functional status after recovery from the acute phase of COVID-19 treated in a hospital setting^31–35^. In the present study, we found that 55.7% of the participants had at least one persistent symptom after one year, and the chance of functional limitation increased fourfold after the same period. A current hypothesis about the effects of long COVID on functional status is that patients continue to have an abnormal immune system due to remnants of a continuous and sustained inflammatory process, even after the acute phase of viral infection. The reasons for this activated inflammation require further investigation, but possibilities include antigen persistence, autoimmune responses by antigenic cross-reactivity, or a damaged repair reflex^27^.

Vaccination against COVID-19 effectively reduces rates of infection and transmission. Evidence also suggests that the incidence of long COVID is reduced among those infected after vaccination. In a study among 28,356 participants in the Office for National Statistics COVID-19 Infection Survey, the likelihood of long COVID symptoms decreased after COVID-19 vaccination, and evidence suggested sustained improvement after a second dose, at least over a median follow-up of 67 days. Furthermore, the authors reported that vaccination may contribute to a reduction in the public health burden of long COVID^36^. However, longer follow-up is needed.

The strengths of the present study are the one-year prospective cohort model after acute COVID-19 infection. In addition, this study included a large number of people who did not require hospitalization, as most studies address the functional status of hospitalized patients. The limitations of this study are as follows: despite sending the Google Forms questionnaire for the data collection to all patients diagnosed as COVID-19 in the municipality during the data collection period, the response rate was very low at 0,67%. This may be because only those with access to the internet and familiarity with digital technology responded, which may explain the selection bias of participants in this study. Participants did not reflect the actual population ratio and were biased toward younger and more educated, mostly female individuals. The lack of a control group made it difficult to infer whether the self-reported symptoms were due to SARS-CoV-2 infection, preexisting comorbidities, or pandemic-related social effects. In addition, the scale used had a subjective score that depended on the patient's perception of their functional status and the prolonged manifestations of COVID-19. Furthermore, the symptoms were self-reported by the participants, so they were subjective according to the perception of each individual.

## Conclusion

After 30 days and one year of the acute infection of COVID-19, patients present functional limitation, according to the PCFS. Although the study population were not hospitalized, it was possible to observe slight to moderate impairment of functionality and associated risk factors include female sex, presence of fatigue, anxiety and depression and at least one persistent symptom after one year. More prospective studies are needed to assess the natural course of acute infection and define the impacts of the post-COVID-19 syndrome. The management of all the effects of the disease requires a greater understanding to implement dynamic and individualized interprofessional interventions.

## Data Availability

The datasets generated and/or analyzed during the current study are not publicly available due to the privacy of participants' personal data but are available from the corresponding author on reasonable request.
